# 5p deletion with congenital diaphragmatic hernia: a case report

**DOI:** 10.1186/s13256-022-03579-1

**Published:** 2022-10-19

**Authors:** Tomomi Kotani, Takafumi Ushida, Noriyuki Nakamura, Kenji Imai, Yukako Iitani, Sho Tano, Shigenori Iwagaki, Yuichiro Takahashi, Miharu Ito, Masahiro Hayakawa, Hiroaki Kajiyama

**Affiliations:** 1 Department of Obstetrics and Gynecology, Nagoya University Graduate School of Medicine, Nagoya, 65 Tsurumai-cho, Showa-ku, Nagoya, 466-8550 Japan; 2 Division of Perinatology, Center for Maternal-Neonatal Care, Nagoya University Hospital, 65 Tsurumai-cho, Showa-ku, Nagoya, 466-8560 Japan; 3 Division of Fetal-Maternal Medicine, Gifu Prefectural General Medical Center, 4-6-1, Noishiki, Gifu, 500-8717 Japan; 4 Division of Neonatology, Center for Maternal-Neonatal Care, Nagoya University Hospital, 65 Tsurumai-cho, Showa-ku, Nagoya, 466-8560 Japan

**Keywords:** Congenital diaphragmatic hernia, Cri-du-chat syndrome, Prognosis

## Abstract

**Background:**

5p deletion syndrome is known as cri-du-chat syndrome, but there are no reports on congenital diaphragmatic hernia complications associated with it.

**Case presentation:**

A 28-year-old primigravida Japanese woman was referred for 5 mm of nuchal translucency. Fetal growth restriction was found at 20 weeks, and a left-sided congenital diaphragmatic hernia was diagnosed at 24 weeks. The karyotype of the fetus was diagnosed as 46, XX, del(5)(p14) and referred to our hospital. At 36^ + 6^ weeks, a 1524 g female infant was delivered after premature membrane rupture, with Apgar scores of 4 and 6 at 1 and 5 minutes, respectively. The baby was intubated immediately with sedation and muscle relaxation, after birth for initial treatment for congenital diaphragmatic hernia. The peripheral blood karyotype was consistent with the prenatal result. The infant was discharged alive, without any respiratory support, after the defect of the diaphragm was repaired.

**Conclusion:**

The results of this study may be helpful for antenatal genetic counseling.

## Background

5p deletion syndrome, one of the most common chromosomal syndromes, was first identified in 1963 by Lejeune *et al*. as cri-du-chat syndrome [Online Mendelian Inheritance in Man (OMIM) number #123450] [1], and is characterized by total or partial deletion (range 35–55%) of the short arm of chromosome 5 [2]. The average size of chromosomal loss in the 5p deletion syndrome is reported to be around 20 Mb (0.6–35.0 Mb) [3]. The breakpoints in most patients have been reported from 5p13 to p15.2 [4], and the critical region is thought to be 5p15.3–15.2 [5]. One of the characteristic features of 5p deletion syndrome in newborns is weak, high-pitched monochromatic crying, which usually disappears in the first year of life. Other features include low birth weight, microcephaly, hypotonia, and severe mental and developmental retardation [2, 3]. Most fetuses show sonographic findings including cerebellar hypoplasia, ventricular septal defect, ventriculomegaly, choroid plexus cyst, and nasal bone hypoplasia [6]. It is estimated that 5p deletion syndrome affects one in every 20,000–50,000 live births [7].

Congenital diaphragmatic hernia (CDH), a diaphragm defect, is a life-threatening anomaly with an incidence of 2–3 cases per 10,000 births [8]. CDH is characterized by intraabdominal organ penetration into the thoracic cavity, leading to pulmonary hypoplasia and hypertension. Recently, there have been improvements in the prenatal diagnosis and evaluation of CDH. However, information on the precise prediction of mortality and morbidity in CDH cases complicated with genetic changes, which is critical for parents, remains limited. Therefore, the outcomes of such rare complications should be accumulated for prenatal counseling.

Herein we present the clinical course of a CDH case complicated with 5p deletion syndrome, which was diagnosed prenatally.

## Case presentation

**Fig. 1.**
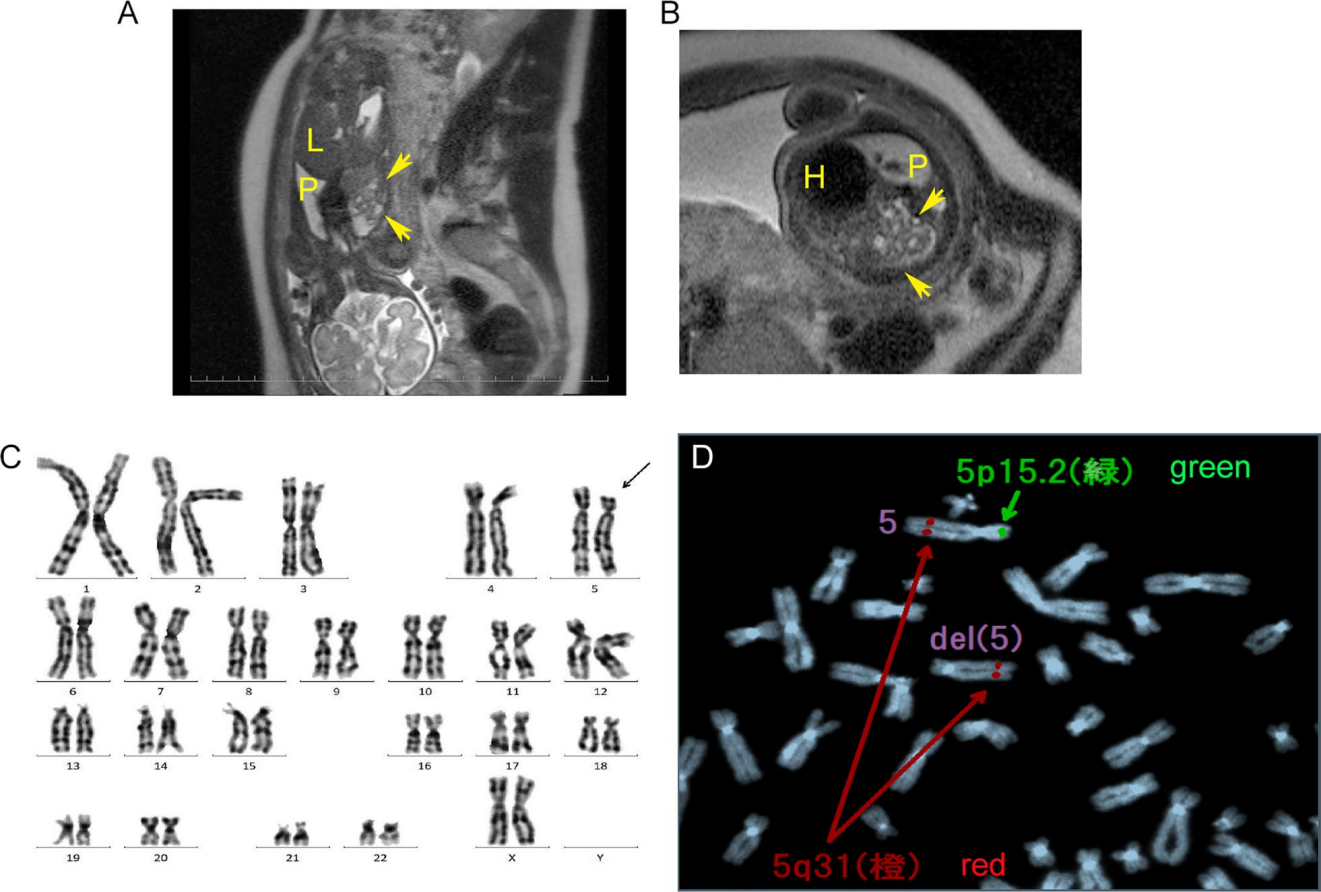
Prenatal evaluation. Fetal magnetic resonance imaging scans in coronal view (**A**) and horizontal view (**B**), at 25 weeks of gestation. Arrows demonstrate protrusion of the intestine. *H* heart, *P* right pulmonary area, *L* liver. **C** The result of Giemsa banding by amniocentesis. The arrow shows an abnormal chromosome 5, as del(5)(p14). **D** The result of fluorescence *in situ* hybridization by amniocentesis. The normal chromosome 5 had both 5p15.2 (green) and 5q31 (red), but no signal of 5p15.2 was detected on the chromosome del(5)(p14)

A 28-year-old Japanese woman was referred for 5 mm of nuchal translucency (NT) at 14 weeks of gestation. She and her partner had a 3-year-old healthy daughter, and the couple had no history of abortion. Following counseling, they did not agree to further prenatal examination. At 20 weeks of gestation, fetal growth restriction was detected, and at 24 weeks suspicious findings of a left-sided CDH and atrioventricular septal defect (AVSD) were observed in the fetus. The observed lung-to-head ratio was approximately 30%. Fetal magnetic resonance imaging revealed intestinal penetration of the thoracic cavity (Fig. 1A, B, arrows), but liver herniation was not detected. Thus, the severity of CDH was estimated as moderate, with a survival rate of 50–60% [9]. Giemsa-banding procedure performed on amniotic fluid aspirated by amniocentesis at 25 weeks of gestation identified the fetal karyotype as 46, XX, del(5)(p14) (Fig. 1B, arrow). In addition, fluorescence *in situ* hybridization (FISH) also revealed the absence of a 5p15.2 signal (Fig. 1C, green spot). After discussion, the parents were determined to provide aggressive treatment to the newborn, who might have been refractory to treatment compared with CDH newborns with normal karyotype.

At 36^ + 6^ weeks of gestation, the pregnant woman was admitted with premature rupture of membranes and labor onset. A 1542 g female neonate was born by spontaneous vaginal delivery without complications. Apgar scores at 1 and 5 minutes after birth were 4 and 6 points (appearance 0 and 1, pulse 1 and 2, grimace 1 and 1, activity 1 and 1, respiration 1 and 1), respectively. Immediately after birth, the baby was intubated with sedation and muscle relaxation according to the standard of care for prenatally diagnosed CDH [10, 11], and was admitted to the neonatal intensive care unit. The mother was discharged 5 days after delivery without complications. The surgery for CDH performed on postnatal day 1 was successful. The evacuated organs were the colon, small intestine, and spleen, and no artificial membranes were needed. The baby was extubated 13 days after birth, but nasal continuous positive airway pressure was required for laryngomalacia. Antenatally suspected AVSD was not detected, but a ventricular septal defect (VSD) was newly found. The patient was transferred to the hospital near her home 42 days after birth, and was discharged without any respiratory support 91 days after birth. At 15 months, the VSD had closed spontaneously, and the infant could not stay sitting up unassisted.

The postnatal karyotyping of peripheral blood was confirmed to be 46, XX, del(5)(p14); however, the karyotypes of both parents were normal. Therefore, the deletion of 5p was determined to be *de novo*. The child also showed the phenotypes associated with 5p deletion syndrome, including developmental impairment.

## Discussion

CDH can be a rare complication associated with 5p deletion syndrome. Several chromosomal anomalies are known to be related to CDH [9]. Importantly, chromosomal abnormalities are often related to poor prognosis of patients with CDH [9]. However, to date, to the best of our knowledge, no data on the prognosis of CDH complicated with 5p deletion syndrome are available on PubMed. Thus, only general information was provided to the couple.

The pathological role of 5p deletion in CDH remains unknown, although few reports have suggested an association between 5p and CDH [12, 13]. For instance, Cornelia de Lange syndrome often associates with CDH, and its most common cause is mutations in the *NIPBL* gene on 5p13.2 [13]. However, the present case retained the 5p13 region. Another patient with CDH presented a 5p15 microdeletion and microduplication of 12p13.3 in a mosaic unbalanced translocation (5;12) [14]. The loss of the 5p15 region is consistent with the present case. In addition, a case of 5p trisomy with bilateral CDH was recently reported, in which the child died of aspiration pneumonia and paralytic ileus at 17 months of age [15]. Recently, part of the 5p deletion syndrome has been reported to be associated with rearrangements between different chromosomes, or with a deletion followed by a duplication in 5p, as revealed by microarray [3, 16]. However, the reported patient’s microarray could not be conducted, and the rearrangements might be relevant to CDH. Thus, accumulation of additional molecularly investigated cases is needed to investigate and further clarify if an association between the 5p region and CDH exists.

## Conclusions

In summary, we present the clinical course of a CDH case complicated with 5p deletion, in which the patient survived and was discharged without any respiratory support after CDH repair. We believe that this study will be helpful in the prenatal counseling of parents of fetuses with similar diagnoses. However, further accumulation of such reports is required.

## Data Availability

Not applicable.

## Abbreviations

AVSD: Atrioventricular septal defect
CDH: Congenital diaphragmatic hernia
VSD: Ventricular septal defect

## References

[1] Lejeune J, Lafourcade J, Berger R, Vialatte J, Boeswillwald M, Seringe P, Turpin R. 3 cases of partial deletion of the short arm of a 5 chromosome. C R Hebd Seances Acad Sci. 1963;257:3098–102. 14095841

[2] Rodríguez-Caballero A, Torres-Lagares D, Rodríguez-Pérez A, Serrera-Figallo MA, Hernández-Guisado JM, Machuca-Portillo G. Cri du chat syndrome: a critical review. Med Oral Patol Oral Cir Bucal. 2010;15:e473-478. 10.4317/medoral.15.e473 20038906

[3] Nevado J, Bel-Fenellos C, Sandoval-Talamantes AK, Hernandez A, Biencinto-Lopez C, Martinez-Fernandez ML, Barruz P, Santos-Simarro F, Mori-Alvarez MA, Mansilla E, *et al*. Deep phenotyping and genetic characterization of a cohort of 70 individuals with 5p minus syndrome. Front Genet. 2021;12: 645595. 10.3389/fgene.2021.645595 PMC8362798 34394178

[4] Mainardi PC, Perfumo C, Cali A, Coucourde G, Pastore G, Cavani S, Zara F, Overhauser J, Pierluigi M, Bricarelli FD. Clinical and molecular characterisation of 80 patients with 5p deletion: genotype–phenotype correlation. J Med Genet. 2001;38:151–8. 10.1136/jmg.38.3.151 PMC1734829 11238681

[5] Wu Q, Niebuhr E, Yang H, Hansen L. Determination of the “critical region” for cat-like cry of cri-du-chat syndrome and analysis of candidate genes by quantitative PCR. Eur J Hum Genet. 2005;13:475–85. 10.1038/sj.ejhg.5201345 15657623

[6] Traisrisilp K, Yanase Y, Ake-Sittipaisarn S, Tongsong T. Prenatal sonographic features of cri-du-chat syndrome: a case report and analytical literature review. Diagnostics (Basel). 2022;12:421. 10.3390/diagnostics12020421 PMC8871365 35204510

[7] Niebuhr E. The cri du chat syndrome: epidemiology, cytogenetics, and clinical features. Hum Genet. 1978;44:227–75. 10.1007/BF00394291 365706

[8] Gallot D, Boda C, Ughetto S, Perthus I, Robert-Gnansia E, Francannet C, Laurichesse-Delmas H, Jani J, Coste K, Deprest J, *et al*. Prenatal detection and outcome of congenital diaphragmatic hernia: a French registry-based study. Ultrasound Obstet Gynecol. 2007;29:276–83. 10.1002/uog.3863 17177265

[9] Deprest J, Brady P, Nicolaides K, Benachi A, Berg C, Vermeesch J, Gardener G, Gratacos E. Prenatal management of the fetus with isolated congenital diaphragmatic hernia in the era of the TOTAL trial. Semin Fetal Neonatal Med. 2014;19:338–48. 10.1016/j.siny.2014.09.006 25447987

[10] Ito M, Terui K, Nagata K, Yamoto M, Shiraishi M, Okuyama H, Yoshida H, Urushihara N, Toyoshima K, Hayakawa M, *et al*. Clinical guidelines for the treatment of congenital diaphragmatic hernia. Pediatr Int. 2021;63:371–90. 10.1111/ped.14473 33848045

[11] Snoek KG, Reiss IK, Greenough A, Capolupo I, Urlesberger B, Wessel L, Storme L, Deprest J, Schaible T, van Heijst A, Tibboel D. Standardized postnatal management of infants with congenital diaphragmatic hernia in Europe: the CDH EURO Consortium Consensus—2015 update. Neonatology. 2016;110:66–74. 10.1159/000444210 27077664

[12] Brosens E, Peters NCJ, van Weelden KS, Bendixen C, Brouwer RWW, Sleutels F, Bruggenwirth HT, van Ijcken WFJ, Veenma DCM, Otter S, *et al*. Unraveling the genetics of congenital diaphragmatic hernia: an ongoing challenge. Front Pediatr. 2021;9: 800915. 10.3389/fped.2021.800915 PMC8852845 35186825

[13] Wynn J, Yu L, Chung WK. Genetic causes of congenital diaphragmatic hernia. Semin Fetal Neonatal Med. 2014;19:324–30. 10.1016/j.siny.2014.09.003 PMC4259843 25447988

[14] Veenma D, Beurskens N, Douben H, Eussen B, Noomen P, Govaerts L, Grijseels E, Lequin M, de Krijger R, Tibboel D, *et al*. Comparable low-level mosaicism in affected and non affected tissue of a complex CDH patient. PLoS ONE. 2010;5: e15348. 10.1371/journal.pone.0015348 PMC3006223 21203572

[15] Nakamura N, Ushida T, Moriyama Y, Imai K, Nakano-Kobayashi T, Osuka S, Goto M, Kajiyama H, Asada H, Hayakawa M, Kotani T. Trisomy 5p with bilateral congenital diaphragmatic hernia: a case report. J Med Case Rep. 2021;15:114. 10.1186/s13256-021-02710-y PMC7945345 33750440

[16] Chehimi SN, Almeida VT, Nascimento AM, Zanardo ÉA, de Oliveira YG, Carvalho G, Wolff BM, Montenegro MM, de Assunção NA, Kim CA, Kulikowski LD. Novel rearrangements between different chromosomes with direct impact on the diagnosis of 5p- syndrome. Clinics (Sao Paulo). 2022;77: 100045. 10.1016/j.clinsp.2022.100045 PMC9160337 35640457

